# Nerve Injuries

**Published:** 1919

**Authors:** Andrè Thomas


### NERVE INJURIES

### Operative Indications Based on the Functional State and the Anatomic Examination of the Nerve

By André Thomas

The therapy of nerve injuries had its period of hesitations and tests before a final solution to the problem was found.

The decisions arrived at during the first months, even during the first years of the war, were often solely based on theory, and the value of the various methods of treatment applied was demonstrated by the results obtained. At present, after the long experience of war, the line of conduct to be adopted in given cases can be suggested by purely practical data.

The personal experience, which we acquired in Professor Dejerine's militarized service at the neurological hospital of the Salpêtrière, was gained during a period when active therapy of nerve injuries began after the cure, at times after cicatrization of the wound. In the various services which we had in our charge at base hospitals, and chiefly at the Salpêtrière, where according to the S. G.'s orders we filled Professor Dejerine's post, we have had to deal solely with nerve injuries dating back to several weeks, sometimes months; in some cases, the fistulization of the wounds was still active and surgical intervention, though necessary from the point of view of the injured nerve, was, for that reason, rendered impossible.

Under the present circumstances, this condition exists no more, for, owing to the improvement brought to surgical technic, primary and secondary suture has been realized, leaving the field open for early surgical treatment of the nerves.

Before deciding whether surgical intervention is needed or not, and in which direction the operation should be performed, a *complete and minute neurological examination* of the patient should take place, giving a precise estimate of the functional value of the nerve. The indirect exploration of the nerve across the teguments, which should be performed whenever possible, and the exploration of the cicatrix or of the cicatricial area are also liable to bring data confirming the diagnosis and giving an idea of the gravity of the lesion.

The clinical examination has been improved by a more careful study of the tissues, distribution of paralysis, atrophy, sensory, circulatory and trophic troubles, of the changes noted in electrical reactions, of the sudoral and pilomotor reactions, etc.

A systematic application to all cases of an equally complete and detailed examination enables the observer to locate the exact site of the lesion, be it in the roots, the plexus, or the large nerve trunks. This site is generally indicated by the very site of the wound, or by the direction of the line running along the latter, from orifice to orifice, but not in cases of multiple wounds situated at a short distance one from the other, where it is important that the wound partially or totally responsible for the nerve injury should be determined.

A careful examination is never to be regretted, for the exact knowledge of whether such and such nervous ramification has been injured or not; whether paralysis is, in a given case, chiefly motor or mainly sensory; whether a given group of muscles and their functions have remained untouched while others are completely paralyzed; whether nerves and muscles are sensitive to pressure, or not, etc., constitutes interesting information from the clinical and physiological standpoints. This becomes invaluable from the point of view of therapy, for, as will be seen later in the course of the present report, before resecting and suturing a nerve, the operator must realize not only what can be gained by it, but also what may eventually be lost.

The symptoms of nerve injuries are grouped according to their distribution, their nature, and their degree of intensity into syndromes corresponding, in schematic cases, to determined lesions. These syndromes have been well described by Professor and Mrs. Dejerine and Mouzon, and may be grouped as follows :

Syndromes of interruption;
" " compression;
" " irritation.

Their knowledge permits a very exact diagnosis of the nature and gravity of the lesions, thus making it possible to give some operative indications.

The above mentioned syndromes are well known, and there is no need for describing them here. However, as the syndrome of interruption, and the value of symptoms liable to be considered as signs of the anatomic interruption of a nerve, have been much discussed, we may state that there is no single symptom permitting one to ascertain during the first few days that the nerve has been sectioned.

The syndrome of physiological interruption, which results in the complete loss of function on the whole area innerved below the lesion, becomes a syndrome of anatomic interruption only after a certain length [of time, ,i. e., once it is permanent. The delay of 3 months, fixed by Mrs. Dejerine, may be adopted as an average.

The syndrome of physiological interruption may be either total or partial. In the latter case, paralysis involves but one group of muscles in the same way as the lesion interrupts only a given number of fascicules. Surgical intervention may be recurred to in both cases, with special measures for each case.

From the clinical standpoint, it is more important to know whether paralysis is *permanent* or *not* for the whole or part of the nerve, and what is the *functional* value of the latter, rather than whether paralysis is partial or total. *Evolution* is an essential element which must be taken into account.

The observer must learn to detect the *first signs of regeneration*: formication produced by pressure on the nerve below the lesion, the pinching test, the pain inflicted to the cutaneous portion of the nerve by pressure on the muscles, paresthesia, reappearance of tonicity, later on the first attempt to contract, etc. These symptoms, which are more easily recognized when the previous examinations have been thorough and complete, constitute, in their turn, a syndrome of regeneration distributed either in the whole nerve or in part of it, according to cases.

The first signs of regeneration are not always the proof that complete functional recovery will follow; they may subside, become limited to an insufficient amount of element, or follow a wrong course in their subsequent development. Once it is ascertained that the improvement will only be slight, surgical intervention is indicated.

In addition to the thorough examination of the functional value of the nerve and the knowledge of the above described syndromes, which permit one to estimate the nature and gravity of the lesion, the nerve itself should be examined whenever possible through the skin. The observer may thus form an exact opinion as to the volume of the nerve, its irregularities, the presence or absence of neuromas, pain resulting from pressure, etc. The value of the information obtained through pressure on the nerve below the lesion has already been mentioned; the presence of formications within the area innerved by the injured nerve shows that the latter still contains some sensitive fibers.

When these manifestations occur in a series of tests, on a gradually extending area, the conclusion may be drawn that the regeneration of the nerve is in progression.

The exploration of either the cicatrix or the skin around the nerve is also necessary, as the presence of synesthesias or topoparesthesias, i. e., sensations in the cutaneous area of the injured nerve, following stimuli applied at the level of the cicatrix, permits one to ascertain that the sheath of the nerve is open, that a number of sensory fibres are interrupted, and that these fibres, during the regeneration process, spread in the neighborhood of the nerve instead of following the course of the latter. This constitutes a double proof that the nerve has been sectioned, and that there is an obstacle to normal regeneration.

From the standpoint of diagnosis, the investigations regarding *the functional value of the nerve, its exploration through the teguments* and *the cicatricial topoparesthesias* are of lesser importance than the *direct anatomic examination.* The latter is readily carried out, and, as it is the preliminary measure in surgical interventions, it should always be performed with a view to confirming diagnosis; in fact the generally recognized diagnostic value of the previously described syndromes was acquired through a perfect co-ordination of the results obtained through anatomic examination with the data furnished by the clinical examinations.

In cases of permanent physiological syndromes of interruption, the nerve is completely sectioned. The upper and lower ends are either quite apart from each other, or joined by a hard, resistant, fibrous cord, or a thick, fibrous block. In the first instance, the upper end of the nerve is more or less neuromatous, while the lower end is slightly rounded; in the second, the fibrous cord or block constitutes a regular cicatricial cheloid connecting the two portions of the nerve. As was demonstrated by histological examination, the cheloid forms an insuperable barrier to neuromatic fibres, and in addition to this both extremities of the sectioned nerve are indurated and fibrous on a given length which varies according to cases.

Is the syndrome of physiological interruption partial? Similar lesions can be found, but the nerve, instead of being sectioned, only bears a more or less deep cut, while a certain number of fibres and fascicules still exist on the opposite side. This cut may be closed either by a fibrous cord or by a cheloid, which thus establishes an apparent uninterrupted connection in the corresponding part of the nerve.

Is it only a matter of compression? The nerve is contained in a fibrous guangue, which, once removed, leaves the nerve more or less flattened out and deformed. Compression is also exerted by an exuberant callus in which the nerve is involved within the bone itself. This is more often observed in the case of the radial nerve because of its close connection with the humerus at the level of the torsion groove.

Finally, in syndromes of simple irritation, the nerve is either pulled by fibrous bridles adhering to the neighboring organs, or it is enlarged and a fibrous nucleus may be felt through the skin. This nucleus varies in length and thickness and enucleates rapidly after opening the sheath. In other cases, the irritation is entertained by a fragment of bone, a foreign body, etc.

In mixed syndromes all[1] the lesions are met with at various degrees of intensity. Several abscesses have been found containing fragments of metal. The nerve may show irregularities, or signs of atrophia, sclerosis, and fibrous nuclei. There is rarely a divergence between the observations resulting from anatomic and clinical examination. However, when such divergence occurs, the question is, as a rule, settled upon second and more careful investigation, sometimes also after surgical intervention.

The *line of conduct* to be adopted is naturally based upon the previous examinations, and varies according to cases, be it total interruption, compression, or simple irritation.

In cases of *total interruption* with or without fibrous link or cheloid, leaving no hope for recovery, suture is indicated after removal of the cheloid and resection of the diseased portion of the nerve, so as to allow the free passage of fibres from the upper to the lower end. However, owing to the considerable interval which, after resection, often separates both ends of the nerve, suture is not always possible; in such cases graft is recurred to, and a nerve taken either from the patient himself or from an animal is inserted.

In cases of *simple compression*, the causative agent is [removed, the fibrous or osseous tissue resected, and the nerve replaced in its normal position. Whenever possible it should be set deeply into fat or muscular tissue after enclosure in a human or animal membrane (in the latter case after special preparation), in order to avoid finding the nerve once more in a fibrous or inflammatory milieu susceptible of reacting on the nervous elements.

1. Causalgia, the pathology of which is more obscure, has been left out here. Various operations have been suggested, from the simple ligature applied immediately above the lesion to the complete section of the nerve. The results obtained varied according to cases. On the other hand, causalgia has a spontaneous tendency to evolve towards recovery within a more or less considerable length of time. A psychic element is often associated with other symptoms, and owing to our ignorance as regards the nature of the lesions and the pathologic physiology of the symptoms, no treatment of causalgia can be recommended. The denudation of the humeral artery was in several instances followed by considerable relief from pain. (Leriche).

The operation is less involved still in cases of *simple irritation*, when the resection of the cicatricial bridles, the opening of the sheath, if the nerve appears tumefiated, and the extraction of a fibrous nodule seem sufficient. This applies not only to the nervous tissues but also to the plexus and the roots.

In the presence of such simple measures, justified by the anatomic examination, one might think that a clinical examination as thorough as that which was previously recommended would be superfluous. This, howewer, is not so, for in a large number of cases the *anatomic exploration alone is insufficient* for deciding upon the line of conduct to be adopted.

If the suture and graft were always successfully performed, one or the other would invariably be recurred to; but it is not the case, and in many a puzzling instance the surgeon has to turn towards the neurologist and leave the decision to him.

We have here, for example, a patient whose median nerve has been partially interrupted; the lateral cut is filled by a cheloid, but the solution of continuity is still maintained by means of some seemingly healthy fascicules. The clinical syndrome is that of a dissociated paralysis. Every section of the nerve, on a given length, always contains the same elements, the fibers serving a muscle or a group of muscles or the sensory fibers. A clinical study of nerve injuries carried out conjointly with an anatomic examination permits one to determine exactly the localization of muscles within the nervous tissues. The idea of suture following resection of the diseased parts comes naturally to the mind, but in a number of cases the distribution of the lesions makes the operation difficult, if not impossible. Should the healthy fascicules be sacrificed in such cases, and complete resection made, followed by complete suture? It is practically impossible to rely on the results of suture in general and especially on those of suture of the median nerve, probably owing to the fact that the latter is a mixed nerve in which the sensory fibers play an active part, due to their number and functional value. This is always a cause of trouble and faulty restoration. It is therefore imperative that a clear estimate of the value of the healthy fascicules should be made, and the observer must have an exact knowledge of the functional value of the nerve, so that in case of a total suture being decided upon the amount to be sacrificed should be fully realized. If the pronator and the palmoris longus are free from paralysis, several fascicules may be sacrificed without great inconvenience, even in the case of partial freedom, in order to perform the suture under favorable conditions. If, on the other hand, the flexion and sensitivity of all fingers or one of them, the forefinger for example, are partially or totally intact, partial suture only should be recurred to.

The preservation of sensitivity is of little importance in injuries of the radial nerve, but such is not the case when the median nerve is concerned. The cutaneous nerves of the fingers are needed as a protection against external traumatisms, circulatory, and trophic troubles. In addition to this, complete functional recovery means not only complete regeneration of the motor fibers of the median nerve, but it also involves the regeneration of the sensory fibers, especially when the forefinger is concerned. This was frequently demonstrated[1].

It is therefore impossible to take general principles as a basis in cases of *dissociated syndromes*, or complicated injuries which cannot be placed under any definite category, and are, anatomically speaking, an assembling of various tissues : partially indurated nerves, cases of atrophia, etc. These are cases for the treatment of which the line of conduct should be based on the *remaining functional value of the nerve*, and on a thorough microscopic examination. The decision to be taken should depend upon joint anatomic and clinical data. Such cases are the best demonstration of the necessity of a close co-operation between medicine and surgery[2].

Similar considerations may be applied to the other nerves. The conservation of a given group of muscles is important in the case of a chiefly motor nerve, such as the radial. The radial muscles do not possess the same functional value as the extensor digitorum. The sciatic popliteal internal may be compared to a certain extent to the median nerve, owing to the sensory innervation supplied. All the cases are not open to discussion ; some injuries to the sciatic above the bifurcation may result in dissociated paralysis of the sciatic popliteals, either external or internal. Suture should, in this case, be partial, and after the ends are joined the healthy portion of the nerve forms a semi-circular branch causing no functional disturbances. This was ascertained in several instances.

In the great majority of cases, *surgical intervention* is recommended, provided the two following conditions are fulfilled :

1. This example is given purposely, for in similar cases surgical intervention was successful, as recorded by Professor and Mrs. Dejerine.

2. Some authors suggested testing the remaining value of the fascicules by mechanical or electrical contraction. Interesting results were thus obtained, furnishing information as regards the anatomic localizations and the section crossed by the nervous fiber of a given muscle, but the correlation existing between the voluntary and direct contractions is not always sufficient, and this method cannot be applied with certainty.

1) anatomic examination of the nerve; 2) surgical treatment of the nerve itself. Every time good results may be expected. The nature of the intervention is determined by the joint data furnished by clinical and anatomic examination, This method of treatment, i. e., close co-operation of the physician and the surgeon, was adopted by Dr. Gosset and his students with full success. We have, on the other hand, always refrained from surgical intervention in cases of severe nervous injuries, incompletely closed wounds with persisting suppuration and fistules, etc. In such cases, surgical treatment was not recurred to until 2 months at least after complete cicatrization of the wound.

Paralysis following injury to the nerve, especially when the *plexus of the upper extremities* is concerned, frequently subsided within several weeks or months without surgical intervention. This, however, cannot be a serious objection to our method. A spontaneously subsiding paralysis is always caused by a very slight lesion of the nerve, which cannot be compared with the severe injuries resulting in permanent paralysis. When treating cases in which a marked improvement was noticeable from week to week, we did not perform any operation. Moreover, all our patients who were operated on were suffering from injuries dating back several months, and the syndromes observed could always be considered as permanent. We are to-day decidedly on the side of surgical intervention. Our reasons are as follows. A simple anatomic exploration of the nerve cannot be harmful. If no lesion requiring a direct intervention on the nerve is discovered, the surgeon refrains from operation; if, on the other hand, adherences, bridles or compression are seen, no matter how slight, the removal of these elements naturally hastens the anatomic and functional restoration of the nerve. Even in cases when recovery might have followed without anatomic exploration or surgical intervention on the nerve, the dressing of the latter certainly contributed to hasten recovery and to make it more complete. We have had the opportunity to operate on patients suffering intermittent pain within the area innerved by a formerly paralyzed nerve. Operation had not been performed in the first instance owing to a gradual attenuation of the symptoms. The anatomic exploration showed the presence of cicatricial bridles. These were excised and a marked improvement in the patient's condition followed. The results would certainly have been better, if intervention had been recurred to earlier. Finally, the examination of wounded whose nerves have been sectioned during the operation, with or without apparent solution of continuity, shows that these lesions do not heal up unaided, and that the signs of regeneration observed in cases when the segments of the nerve are joined by some fascicules are very limited and never reach a sufficiently high standard from the functional standpoint.

The advisability of *exploratory* and *therapeutic intervention* is no longer open to discussion. The regenerative power of neuromas is undoubtedly great, as can be seen from the luxuriant growth of branches in neuromas, but it is nevertheless limited and decreases gradually. As was demonstrated some time ago in the study of nerves and nervous centres of amputated patients, a certain amount of fibers from the central branch are subject to retrograding atrophia which increases gradually. The cells of the anterior branches, the fibers of which have been sectioned, are subject to lesions liable to be followed by atrophia or total disappearance. For this reason, *early surgical treatment* of the nerve is recommended.

Nerve surgery itself enters into a new era, owing to the fact that the early treatment of wounds has been considerably improved of late. The early disinfection and sterilization of wounds, which makes it possible to perform primary and secondary suture, permits one to proceed to the anatomic examination of nerves much sooner, and to perform the immediate suture of same whenever a severe lesion is discovered.

Although we have no personal experience in this respect, we have every reason to believe that the results obtained after such an intervention are far more satisfactory than they would have been, had the nerve been previously submitted to a more or less prolonged infection. The information coming to us from various sources only confirms the above statement. When the condition of the nerve is such that the desirability of immediate suture is open to question, the nerve should be merely cleaned and disinfected, and the operation postponed until the necessity for it becomes obvious. All patients should be evacuated to a neurological center and a card bearing the nature of the operation performed (if any), and data regarding the anatomic condition of the nerve attached to their medical records. We are unfortunately unable to give any statistics supporting the above expressed opinion. We have also been unable to keep full records of many of the patients on whom suture has been performed. In a large number of cases the operation was carried out under very unfavorable conditions, after long suppuration; the nervous sections joined by suture were not absolutely healthy. On the contrary, excellent results were recorded when both ends of the nerve were normal. According to our own experience, and to that of a number of surgeons and neurologists, the sutures of the radial nerve are far more satisfactory than those performed on the cubital and median nerves. However, in one instance the suture of the median nerve, carried out a year after the wound was inflicted, resulted in complete regeneration, both functional and sensory. No sign of recovery was noted before operation was performed, and complete restoration followed within a year.

The above observation applies not only to the nerves but also to their roots. A case of radicular paralysis of the brachial plexus is reported, in which the patient was able to contract the deltoid, the biceps, and the spinous muscles 18 months after suture of the fifth and sixth cervical roots. Similar satisfactory restorations have been observed in sutures of the sciatic nerve. These examples are very convincing, the more so because suture was always performed a few months, even more than a year, after the wound, and no sign of regeneration was observed before the operation. Without surgical intervention the nerve would have remained damaged, and complete paralysis would have ensued. A large number of exemples illustrating the necessity for early suture have been published of late by various neurologists and surgeons, especially by Professor and Mrs. Dejerine, who, since the beginning of the war, have strongly recommended this method.

*Graft* should not be recurred to except in cases where no other measure is possible. Out of the large number of grafts performed by Doctor Gosset, only two instances of partial recovery have been reported. The nerve to be grafted was always taken from the patient. It was as a rule a musculo-cutaneous nerve from the lower part of the leg. In the two recoveries noted (in both cases the cubital nerve was concerned) the results obtained were as follows : (1) The anterior cubital muscle contracted normally; the flexion of the two last fingers progressed satisfactorily; and the general condition improved. (2) The cubital nerve had also been sectioned and the lower third of the arm ; the anterior cubital and the flexus longus of the two last fingers contracted two and one-half years after graft was performed, and a contraction was observed in the muscles of the hypothenar eminence.

We have not had the opportunity of making use of hetero-graft, i. e., the nerve of an animal preserved in formol or alcohol, which has given such remarkable results in Doctor Nageotte's experiments, but the value of which has not yet been ascertained in the treatment of men. We do not believe that graft is favorable to regeneration by supplying sheaths to the new branches. It seems to us that graft is acting chiefly as a line of conduct to the regenerated fibers.

The results obtained in nerve surgery by the use of graft have so far been far less satisfactory than those given by suture. Theoretically graft has the advantage of being fixed on both ends of the nerve after resection of the diseased tissues. In practice, however, graft is performed chiefly in cases when the interval between the two ends of the nerve is of several centimetres, which constitutes in itself an unfavorable condition to regeneration. On the other hand, *suture* is promising whenever the portions of the nerve are absolutely healthy, i. e., when they have the consistency and the aspect of a normal nerve, when bleeding is abundant, and when the segments of fibers meet exactly. For this reason early suture performed on nerves which have not yet been deformed and separated by superficial bridles has more chances of success than that carried out later. Late sutures always imply a danger of faulty regeneration, of interchange between motory and sensory fibers, and between the fibers of various muscles, thus giving rise to syncinesis and parakinesis. A faulty regeneration always hampers functional recovery. The exact apposition of corresponding sections of two nervous segments is not an easy task. For this reason resection of the fascicules which have remained joined is often carried out in order to perform complete suture. Before resection a thread must be tied either above or under the lesion so as to permit the exact juxtaposition of the two segments concerned.

The results achieved by simple liberation and decompression have been so completely successful and numerous that there is no need to come back on this point.

Surgical treatment does not necessarily do away with other methods: massage, electric treatment, and early mobilization, which is a sure remedy against articular ankylosis, circulatory troubles, etc. Special attention must be paid to the first symptoms of contraction, so that adequate measures should be taken for the motor and sensory reeducation. All *orthopedic apparatus* which is useful during the period of complete paralysis of certain nerves becomes frequently harmful during the period of restoration, for it favors the development of antagonistic forces, the inertia of paralyzed muscles, etc. As soon as mobility of the organ returns, it is advisable to use orthopedic apparatus as little as possible.

The above observations may be summarized by the following formula, which corresponds to the opinion of the great majority of neurologists and surgeons. *In cases of nerve paralysis following*

*injury, an anatomic examination must always follow and complete the clinical examination. The operation to be performed is decided upon according to the joined data furnished by the physician and the surgeon.*

(The following articles by Dr. André Thomas may also prove of interest.)

Paralysies multiples des nerfs craniens. Troubles sensitifs dans le domaine du trijumeau et du plexus cervical. Plaque de pelade dans le territoire de la branche mastoïdienne du plexus cervical. *Société de Neurologie*, July, 1915.

Hypertonie musculaire dans la paralysie radiale en voie d'amélioration. Sensations cutanées dans le domaine du nerf radial provoquées par la pression des muscles qui reçoivent leur innervation du même nerf. Égarement des cylindres-axes régénérés, destinés à la peau, dans les nerfs musculaires. *Société de Neurologie*, July 29, 1915.

La forme de la contraction idéo-musculaire dans les lésions des nerfs périphériques. La décontraction lente, mécano-réaction de dégénérescence. *Société de Biologie*, January 22, 1916.

Résection et suture de la VI[e] racine cervicale, dans un cas de paralysie radiculaire supérieure du plexus brachial. *Société de Neurologie*, February 3, 1916.

Restauration défectueuse des fibres sensitives. Topoparesthésies. Synesthesies. *Société de Neurologie*, February 3, 1916.

Hypermyotonie ou contracture secondaire dans la paralysie du nerf médian par blessure de guerre. *Société de Neurologie*, June 29, 1916.

Paralysie radiculaire supérieure du plexus brachial. Innervation radiculaire du C V et du C VI. Examen du tonus. *Société de Neurologie*, June 29, 1916.

Topoparesthésies cicatricielles. Examen des troncs nerveux et des cicatrices dans les blessures des nerfs. *Paris Médical*, June, 1916.

Variations et réactions. Hémorragies locales dans les blessures du système nerveux. *Société de Biologie*, October 21, 1916.

Hyperidrose par irritation périphérique. *Société de Neurologie*, November 9, 1916.

Des erreurs d'aiguillage dans la restauration des fibres motrices. Parakinésies. Syncinésies. Synergies paradoxales. *Paris Médical*, July, 1917.

Syndrome sympathique radiculaire et causalgie. *Société de Biologie*, November 24, 1917.

Le tonus du poignet dans les paralysies du cubital. *Paris Médical*, December 1917.

Sur le phénomène du pincement de la peau dans les blessures des nerfs périphériques. *Société de Biologie*, November 24, 1917. (En collaboration avec MM. Lévy, Valensi et Courgeon).

Sur un cas de restauration rapide après suture du nerf médian. *Société de Neurologie*, December, 1917.

* The titles marked with an asterisk have been taken from the original papers.

